# Hyperspectral and Multispectral Image Fusion with Automated Extraction of Image-Based Endmember Bundles and Sparsity-Based Unmixing to Deal with Spectral Variability

**DOI:** 10.3390/s23042341

**Published:** 2023-02-20

**Authors:** Salah Eddine Brezini, Yannick Deville

**Affiliations:** 1 Institut de Recherche en Astrophysique et Planétologie (IRAP), Université de Toulouse, UPS-CNRS-CNES, 31400 Toulouse, France; 2 Laboratoire Signaux et Images, Université des Sciences et de la Technologie d’Oran Mohamed Boudiaf, Bir El Djir, Oran 31000, Algeria

**Keywords:** spectral variability, spectral unmixing, hypersharpening, fusion, hyperspectral/multispectral image, spectra bundles, sparse unmixing, automated extraction of endmember bundles, sparse regression based unmixing

## Abstract

The aim of fusing hyperspectral and multispectral images is to overcome the limitation of remote sensing hyperspectral sensors by improving their spatial resolutions. This process, also known as hypersharpening, generates an unobserved high-spatial-resolution hyperspectral image. To this end, several hypersharpening methods have been developed, however most of them do not consider the spectral variability phenomenon; therefore, neglecting this phenomenon may cause errors, which leads to reducing the spatial and spectral quality of the sharpened products. Recently, new approaches have been proposed to tackle this problem, particularly those based on spectral unmixing and using parametric models. Nevertheless, the reported methods need a large number of parameters to address spectral variability, which inevitably yields a higher computation time compared to the standard hypersharpening methods. In this paper, a new hypersharpening method addressing spectral variability by considering the spectra bundles-based method, namely the *Automated Extraction of Endmember Bundles* (AEEB), and the sparsity-based method called *Sparse Unmixing by Variable Splitting and Augmented Lagrangian* (SUnSAL), is introduced. This new method called *Hyperspectral Super-resolution with Spectra Bundles dealing with Spectral Variability* (HSB-SV) was tested on both synthetic and real data. Experimental results showed that HSB-SV provides sharpened products with higher spectral and spatial reconstruction fidelities with a very low computational complexity compared to other methods dealing with spectral variability, which are the main contributions of the designed method.

## 1. Introduction

The continuous progress of remote sensing sensors allows one to have a better understanding of the different phenomena surrounding us [1]. In particular, the remote sensing hyperspectral images acquired by high-spectral-resolution sensors consist of hundreds of contiguous spectral bands ranging from the visible to infrared wavelength domains. The hyperspectral sensors can be either onboard spaceborne platforms including EO-1/Hyperion [2], AVIRIS [3], PRISMA [4], HISUI [5], EnMAP [6] or onboard aircrafts equipped with such sensors as Hyspex [7], AVIRIS-NG [8], and APEX [9]. The data provided by these sensors having a high spectral resolution deliver useful information that enable an accurate classification and precise detection of pure materials (also called endmembers) in the observed scene. This fine spectral resolution permits the use of hyperspectral images (HSI) in countless different fields [10] including monitoring of coastal areas [11,12], measuring gas flaring [13,14], estimation of the area of photovoltaic panels [15], mineral detection, and mapping [16].

However, because the acquisition of HSIs is realized in narrow bandwidths, it constrains the remote sensing hyperspectral sensors to operate in such a way as to achieve an optimal trade-off between satisfactory signal-to-noise ratio (SNR) [17] and spatial resolution. In other words, the remote sensing hyperspectral sensors must obtain enough photons to retain an acceptable SNR [18]. This physical limitation provides HSIs with low spatial resolution and consequently hinders their use [19] in applications requiring both high spectral and spatial resolutions like classification or vegetation monitoring.

A straightforward manner to circumvent such a limitation is to fuse an HSI with a multispectral image (MSI) of the observed scene, acquired approximately at the same time. As a matter of fact, the MSIs exhibit a high spatial resolution compared to the HSIs and have a low spectral resolution (they are acquired over at most around ten spectral bands). Ideally, the main objective is to provide an unobserved high-spatial-resolution hyperspectral image by using the spatial information contained in the MSI while preserving as much as possible the spectral fidelity of HSI. This fusion process is known as hypersharpening [20] and can be seen as an extension of pansharpening which consists of merging a panchromatic image (PAN) with an MSI or HSI. Nevertheless, pansharpening appears more complex to achieve compared to hypersharpening due to the significant gap between the spectral domains covered by the PAN and HSI images [21].

Various hypersharpening approaches have been developed and among them ones using a Bayesian formulation [22,23,24]. Recently, a novel hypersharpening scheme was introduced and tested on WordlView-3 data [25]. Tensor representations have also been considered for the fusion process [26,27,28,29,30]. Other methods are based on sparse regression [18,19]. Currently, Deep Learning (DL) techniques are extensively used for hypersharpening. Some techniques are based on *Convolutional Neural Networks* (CNN). In [31], the authors proposed a *Spatial-Spectral Reconstruction Network* (SSR-Net) trained by optimizing both spatial and spectral edge losses. In [32], a new loss function called *RMSE, angle and Laplacian* (RAP) to reduce the spectral-spatial distortions was introduced. Even though the CNN methods prove their effectiveness, these techniques are not always suitable for real scenarios [33]. Indeed, these networks are trained on simulated data, the *Spectral Response Function* (SRF) and *Point Spread Function* (PSF) are required to be known and generally are not always available in practical real scenarios. To overcome this problem in [34], the authors explicitly take into consideration the spectral low rank of the HSI. Other techniques consider *Generative Adversarial Networks* (GAN). In [35], an improved *Super-Resolution* GAN (SRGAN) was applied to remote sensing images. The GAN based methods are generally subject to spectral-spatial distortions due to the mode collapse inherent to the GAN [36,37]. In [37], a *Latent Encoder Coupled* GAN (LE-GAN) was proposed to improve the spectral-spatial fidelity of the fusion products. For more details, the reader can refer to the recent review devoted to DL based techniques for image fusion in [33].

Considerable emphasis has been put on methods based on *Spectral Unmixing* (SU) intended for hypersharpening [38,39,40,41]. Such approaches aim to extract the spectral information (the spectral signature of the endmembers) contained in the HSI and the spatial information (the abundance coefficients) included in the MSI. To this end, they employ the techniques developed in the field of *Blind Source Separation* (BSS), especially those using the *Nonnegative Matrix Factorization* (NMF) framework [42,43,44]. Most of these SU methods are based on the *Linear Mixing Model* (LMM) [1] mainly due to its simplicity. In particular, this model assumes that each endmember is described by only one spectral signature in the whole image. However, this assumption is no longer valid when some physical phenomena occur in the observed scene. Thus, the LMM appears rather limited by two main issues, namely: the spectral/intra class variability and the nonlinearity [45,46,47].

Currently, a growing attention is dedicated to tackle the spectral variability by introducing the notion of class of endmembers instead of the concept of endmembers. Several methods have been developed to this end, particularly using parametric models [47]. These models aim to integrate the spectral variability directly in the LMM, like in [48,49,50] which incorporate additive terms in the LMM or using scaling factors [51,52,53]. The nonlinearity can be caused by multiscattering effects or intimate interactions [54]. To overcome this limitation, authors in [55,56,57] have proposed a *Linear Quadratic* NMF (LQ NMF) or a Bilinear NMF. Models and algorithms employed for nonlinear unmixing are described in [54]. Indeed, neglecting the spectral variability and nonlinearity may spread error during hypersharpening and particularly the spectral variability. The present paper aims at addressing the spectral variability issue. Therefore, several methods based on SU have been investigated to tackle the spectral variability in the fusion process [28,30,58,59,60]. The cited methods provide hypersharpening products with an interesting spectral and spatial fidelities. However, these techniques present a high computational complexity particularly the methods based on parametric models. Indeed, they need a large number of parameters and variables to address spectral variability. To reduce the computational load of the above cited approaches, we propose a new hypersharpening method using a sequential strategy. The introduced method is based on spectra bundles (which are composed of the extracted set of spectral signatures) [61,62].

The introduced method called Hyperspectral Super-resolution with Spectra Bundles dealing with Spectral Variability (HSB-SV) considers the Automated Extraction of Image-Based Endmember Bundles or Automated Extraction of Endmember Bundles (AEEB) [61]. The AEEB method is a simple and efficient way to handle the spectral variability by building a spectral library from the pure material spectra extracted from the HSI. This enables one to construct a spectral dictionary compatible with the physics of the HSI as the candidate endmembers are estimated directly from the considered HSI. Moreover, the use of the AEEB method significantly reduces computational complexity. Then, to estimate the high-spatial-resolution abundance maps, a sparse regression technique is considered, namely Sparse Unmixing by Variable Splitting and Augmented Lagrangian (SUnSAL) [63]. The SUnSAL technique is applied using the down-sampled candidate endmember spectra and the MSI. Finally, the fusion product is obtained by combining the extracted high-resolution pure material spectra and the high-spatial-resolution abundance maps.

The main contributions of this paper are as follows:

- Significantly reducing the processing time with respect to the hypersharpening methods addressing spectral variability.
- Solving the hypersharpening problem by deriving a spectral library and applying a sparsity-based method to improve the spatial and spectral fidelities of the hypersharpening products.
- Dealing with multiple types of spectral variabilities like illumination variations and intrinsic variability or caused by other phenomena since the physics of the considered scene is respected in the proposed approach by using the spectral signatures extracted directly from the considered HSI.

The remainder of the paper is structured as follows. Section 2 is devoted to the related works. In particular, the recent techniques incorporating the spectral variability in the fusion process by employing parametric models are described. The observation model based on the LMM and details of the proposed hypersharpening approach are introduced in Section 3. The Section 4 describes the synthetic and real data used for all the conducted experiments. In Section 5, the experimental results based on synthetic and real data are presented. The results of the designed approach are compared to those provided by some of the state-of-the-art methods, in particular the recent methods tackling spectral variability. Finally, Section 6 concludes this paper.

## 2. Related Works

In this section, some recent spectral unmixing hypersharpening techniques addressing spectral variability by means of parametric models are reported. The spectral variability is often induced by several factors such as:

- Illumination changes, mainly caused by topography variations in the observed scene affecting the angles of the incident radiation.
- Atmospheric conditions which alter the radiance measured by the hyperspectral sensors.
- Intrinsic spectral variability caused by physicochemical differences especially in observed scenes constituted by vegetation.

The FuVar method [58] for *HS-MS Image Fusion with spectral Variability*, addresses the spectral variability in the case of seasonal spectral variability (inter-image). More precisely, the FuVar method considers a parametric model called the *Generalized* LMM (GLMM) [64]. The GLMM model translates the spectral variability as scaling factors that depend both on the pixel and the spectral band. This provides the GLMM the flexibility to handle the spectral variability. The GLMM is generally adopted for spectral variability caused by the illumination changes and seasonal changes [58]. The FuVar method considers the *Alternating Direction Method of Multipliers* (ADMM) to solve the fusion problem. Furthermore, the FuVar method appears very effective when fusing HSI and MSI with spatially uniform variations. However, the spectral variability cannot only be described by illumination and topography changes since spectral variability can be induced by several factors. Furthermore, methods based on parametric models (like FuVar) require substantial user supervision for tuning the involved parameters, which is a challenging task when it comes to addressing non-convex problems. Moreover, the FuVar method has a high computational cost since it takes in consideration a large amount of variables.

A recent approach addresses the spectral variability to merge HSI with MSI acquired approximately at the same time. This approach is known as *Hyperspectral and Multispectral data fusion based on IP-NMF* (HMF-IPNMF) [59]. This method applies the *Inertia-Constrained Pixel-by-Pixel* NMF (IP-NMF) [65] to extract, for each class of endmembers, slightly different spectral signatures for each pixel of the HSI. The IP-NMF method proves to be very attractive when it comes to handle spectral variability arising from intrinsic variability caused by physicochemical differences [12,65]. Furthermore, unlike the *Coupled NMF* (CNMF) [38] or *Joint-Criterion NMF* (JCNMF) [40] which use alternating or joint iterative algorithms, HMF-IPNMF considers a simple sequential strategy composed of three main stages. The first one is the extraction of hyperspectral endmember spectra via IP-NMF [65]. The second stage consists of estimating high-spatial-resolution abundance fractions through a linear regression, using the *Fully Constrained Least Square* (FCLS) method [66]. The last stage combines both results of the first and second stages to obtain the fusion product. Nevertheless, HMF-IPNMF uses specific matrix structures involving many variables to describe the HSI, which leads to a significant processing time.

A recent hypersharpening technique [67] extends the JCNMF [40] method to handle the spectral variability by exploiting the same specific matrix structure of the used matrices in IP-NMF [65]. The JCNMF method, contrary to CNMF, simultaneously unmixes the HSI and MSI. Moreover, JCNMF exploits the spatial degradation between the MSI and HSI. The degradation operator can be considered as a blurring-decimation matrix containing Gaussian filter values. Ideally, this operator can represent the PSF. This degradation model is used to generate realistic synthetic data in the conducted experiments. Nonetheless, this joint unmixing implies that not only the HSI but also the MSI is described by the above-mentioned specific structures of matrices which inevitably increases the number of variables and leads to a high computational time.

Another method accounting for spectral variability and called FSVA [60] has also been proposed. Like FuVar, FSVA is based on the parametric model known as the *Augmented* LMM (ALMM) [53] and solves the fusion problem by using an alternating strategy (ADMM). The ALMM is an extension of the *Extended* LMM (ELMM) [68] obtained by adding a low rank term to the ELMM to describe more complex spectral variability. This feature permits FSVA to simultaneously handle the scaling factors, intrinsic variability and nonlinearity, which leads to performance improvement. Furthermore, FSVA tries to combine a spatio-spectral degradation model and the spectral variability model. As for SU based methods dealing with spectral variability, it requires a large number of variables leading to a high processing time.

## 3. Proposed Approach

### 3.1. Observation Model

For a proper understanding, we summarize the required principles of LSU [1] in this section because the proposed method circumvents one of the main limitations of LSU namely the spectral variability. The LSU assumes that any observed pixel of the HSI or MSI corresponds to a linear mixture between the endmember spectra, weighted by the associated abundance coefficients following the observation models

(1) $$X_{h}=S_{h}A_{h},$$

(2) $$X_{m}=S_{m}A_{m},$$

where $X_{h}\in R_{+}^{L_{h}\times P_{h}}$ and $X_{m}\in R_{+}^{L_{m}\times P_{m}}$ are the observed hyperspectral and multispectral images, respectively; P stands for the number of pixels and L the number of spectral bands, with *h* and *m* indices referring to the hyperspectral and multispectral images, respectively; $A_{h}\in R_{+}^{N\times P_{h}}$ and $A_{m}\in R_{+}^{N\times P_{m}}$ are the spatially degraded associated abundance fractions of the HSI and associated abundance fractions of the MSI; N represents the number of endmembers. For the sake of clarity, N is assumed to be the same for both HSI and MSI. The estimated hyperspectral and spectrally degraded multispectral endmember spectra are denoted by $S_{h}\in R_{+}^{L_{h}\times N}$ and $S_{m}\in R_{+}^{L_{m}\times N}$.

The spatially degraded abundances $A_{h}$ and the spectrally degraded endmember spectra $S_{m}$ are here modelled as

(3) $$A_{h}=A_{m}F,$$

(4) $$S_{m}=RS_{h}.$$

The matrix $F\in R_{+}^{P_{m}\times P_{h}}$ represents the *Point Spread Function* (PSF) and $R\in R_{+}^{L_{m}\times L_{h}}$ the *Spectral Response Function* (SRF). These two functions have a significant role during the fusion process to respect the physics. Indeed, to preserve the physical meaning of the fusion process, we must consider the sensor spectral response for each band [69]. It is not physically meaningful if the SRFs of the considered sensors for the fusion do not overlap [69]. Furthermore, the hypersharpening aims to provide a fused product from an ideal virtual sensor that would combine the spectral sensitivity of the hyperspectral sensor and the high spatial resolution of the multispectral sensor [69].

### 3.2. Description of HSB-SV

As mentioned above, the proposed approach is based on a sequential strategy used in HMF-IPNMF framework. It is divided in three main parts: (1) Estimation of the Hyperspectral Endmember Spectra by employing the AEEB method; (2) Estimation of High-Spatial-Resolution Abundance Fractions by means of the SUnSAL method; and (3) Fusion stage.

#### 3.2.1. Extraction of Spectral Library by AEEB

The first part of the introduced technique aims to estimate endmembers from HSI by considering the spectral variability. More precisely, the objective is to extract a spectral dictionary. The main motivation behind the use of a spectral library is mainly due that it can deal with different types of spectral variabilities. Indeed, hypersharpening algorithms based on parametric models like FuVar rely on the assumption that the spectral variability can be described by only considering scaling factors, which is not always relevant. The most significant example showcasing this is an HSI describing urban areas composed by various pure materials including vegetation (green spaces), tile roofs, architectural monuments, small streets, etc. In this case [65], the spectral variability is arising from different causes. Building a spectral library directly from HSI allows one to handle the spectral variability efficiently and effectively by considering different factors. Constructing a spectral dictionary from a HSI is a simple task which consists of applying an *Endmember Extraction Algorithm* (EEA) [1] to random subsets of the HSI to obtain multiple signatures of each pure material in the observed scene. Moreover, using EEA techniques reduces undoubtedly the computational load compared to the hypersharpening methods cited in Section 2. Basically, the AEEB method is built on the assumption that the statistics of the HSI can be approximately recovered with a small fraction of it [61]. In other words, if enough pure pixels are present in the HSI then they will be available in the randomly selected subset from that HSI [61,70]. The validity of such an assumption relies on the size of the subsets and the number of pure pixels present in the observed scene [61].

Due to its simplicity, the AEEB method allows one to have an efficient representation of the spectral variability with a very low computational cost. Recent methods proposed to directly incorporate the spectra bundles (which are composed of the extracted set of spectral signatures for each run and for each class of endmembers) into the LMMs [71]. The endmember bundles can be expressed as [71]

(5) $$B=[B_{1}|B_{2}|\cdots |B_{j}]$$

where $B_{j}\in R_{+}^{L_{h}\times Y_{j}}$ denotes the bundle representing the j-th class, J is the number of classes, $Y_{j}$ is the number of pure spectra in the j-th class and Y the total number of endmember spectra of all classes with $Y=\sum_{j=1}^{J}Y_{j}$.

Thus, an observed hyperspectral pixel spectrum $x_{h_{i}}\in R_{+}^{L_{h}\times 1}$ is expressed as

(6) $$x_{h_{i}}=Ba_{h_{i}}$$

where $a_{h_{i}}\in R_{+}^{Y\times 1}$ stands for the abundance coefficients corresponding to each individual spectrum of the endmember bundles B.

Furthermore, to apply the AEEB method some parameters must be fixed *a priori* like the number of pure materials present in each subset, the number of subsets and then their sizes. The number of pure materials represents the number of endmembers present in each subset. The number of subsets corresponds to the number of subsets randomly selected from the HSI that is used by the AEEB method to provide the spectral library. The size of each subset is the number of pixels. The performance of the AEEB method (and the methods based on a spectral library) is related crucially to the presence of sufficient number of pure pixels in the HSI to have a coherent description of the spectral variability present in the scene. For all the conducted experiments, we use the well-known *Vertex Component Analysis* (VCA) [72] method as EEA. Indeed, VCA is a fast EEA which also permits to reduce the processing times.

The complete algorithm of HSB-SV is described in Algorithm 1.
**Algorithm 1.** Hyperspectral Super-resolution with Spectra Bundles dealing with Spectral Variability (HSB-SV).**Input:** hyperspectral image $X_{h}$ and multispectral image $X_{m}$.
**Output:** the unobservable sharpened high-spatial-resolution hyperspectral image $\tilde{X_{f}}$.
Set the number of pure materials.Set the number of subsets representing the number of applied runs of AEEB.Set the size of subsets used by the AEEB method.Extract B from $X_{h}$ by running AEEB over the selected number of subsets.Deduce $B_{m}$ by downsampling B using (4).Obtain $a_{m}{}_{i}$ by solving (7) using SUnSAL.Recombine B and $a_{m}{}_{i}$ by using (8) to obtain $\tilde{X_{f}}$.


#### 3.2.2. Estimation of High-Spatial-Resolution Abundance Fractions

The second stage of the introduced method aims at extracting the high-spatial-resolution abundance fractions (stored in $A_{m}$) in each pixel individually from the MSI. To this end, the sparsity regression-based method called SUnSAL [63] is applied. The main goal of sparse regression-based techniques is to estimate abundance coefficients from a large spectral library already available. Indeed, a small number of endmembers are active in a given pixel. Therefore, the sparse unmixing allows one to obtain a linear combination of pure material spectra for each of the observed remote sensing spectra. In other words, the sparse unmixing tries to estimate the optimal subset of pure materials in the spectral library than can best represent each mixed pixel in the observed scene [1]. Moreover, sparse unmixing methods are generally efficient in terms of computational cost [47]. This feature decreases quite significantly the processing time of HSB-SV especially compared to the hypersharpening methods addressing spectral variability. The performance of sparse unmixing methods relies on the availability of suitable spectral libraries [1]. In our case, the spectral library was extracted directly from the HSI and consequently allows to improve the performance of the SUnSAL method.

For each multispectral pixel $x_{m_{i}}$, the high-spatial-resolution abundance fractions associated $a_{m}{}_{i}$ (forming part of $A_{m})$ associated with the i-th pixel is estimated by means of the SUnSAL method, which is used between the multispectral image $X_{m}$ and the multispectral spectra bundles forming the matrix $B_{m}$. The matrix $B_{m}$ is derived from B extracted in the first stage in the same way as in (4) using the SRF of the considered sensors in the experiments. The SRF can be known or estimated. Indeed, only a few instances of the dictionary $B_{m}$ are used to reconstruct a pixel spectrum. The objective of the SUnSAL method is to estimate the high-spatial-resolution abundance fractions $a_{m}{}_{i}$ by optimizing the following cost function, separately for each pixel

(7) $$\underset{a_{m}{}_{i}}{\min\;}∥B_{m}a_{m_{i}}-x_{m_{i}}{∥}_{2}^{2}+\lambda ∥a_{m_{i}}{∥}_{1}$$

where $∥\cdot {∥}_{2}^{2}$ is the $\ell_{2}$-norm and $∥\cdot {∥}_{1}$ is the $\ell_{1}$-norm which is responsible for promotingsparsity. λ is a non-negative parameter which tunes the relative weight between the $\ell_{1}$ and $\ell_{2}$ terms of (7).

The SUnSAL method makes use of the ADMM to optimize (7). As in the first stage of our method, the main motivation behind applying the SUnSAL method is that it can deliver efficient results with low computational complexity.

#### 3.2.3. Fusion

The third and last stages of the proposed approach consists of creating the fusion product $\tilde{X_{f}}$ by recombining the obtained matrices. Hence, each pixel spectrum $\tilde{x_{f_{\dot{i}}}}$ of the unobservable sharpened high-spatial-resolution hyperspectral image $\tilde{X_{f}}$ is defined as

(8) $$\tilde{x_{f_{\dot{i}}}}=Ba_{m_{i}}$$

## 4. Datasets

For the conducted experiments, two sets of data were considered, namely synthetic and real data. We chose data with a small size because HMF-IPNMF and FuVar involve important memory capacity and computation cost as these two methods use large size matrices as suggested by the authors of the corresponding methods [58,59].

### 4.1. Synthetic Data

**Figure 1:**
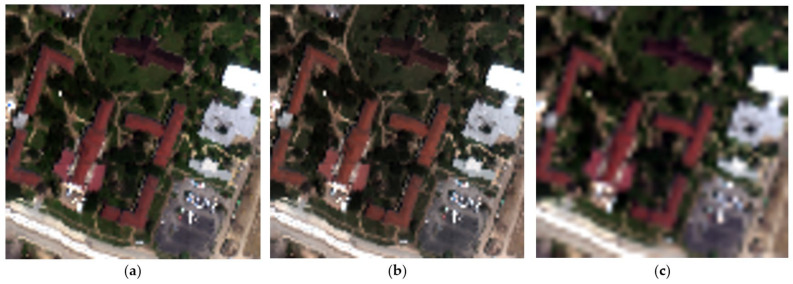
True-color image composite for the synthetic dataset. (**a**) Original Hyperspectral image; (**b**) Low-spectral-resolution multispectral image; (**c**) Low-spatial-resolution hyperspectral image.

The synthetic data sets were obtained from a real airborne high spatial and spectral resolution hyperspectral image [73]. This real hyperspectral image covers the spectral domain 0.35–1.05 μm with 144 wavelengths. More precisely, we used a subset (Figure 1) of this hyperspectral image with 100×100 pixels [59]. This subset was constituted by seven classes of endmembers with spectral variability. The selected subset was used to obtain synthetic data sets, more precisely to create two synthetic images by means of Wald’s protocol [74]. To this end, the subset was spatially and spectrally degraded to obtain the low-spatial-resolution hyperspectral image and the low-spectral-resolution multispectral image respectively. The low-spatial-resolution hyperspectral image was generated by degrading the real hyperspectral image by a factor of 2. This degradation was applied by considering a blurring-decimation matrix with a Gaussian filter like in [40] (this can represent the PSF in (3)). The low-spectral-resolution multispectral image was created by spectrally degrading the original image by using the ENVI software and considering the SRF of the QuickBird sensor (Table 1).

### 4.2. Real Data

**Figure 2:**
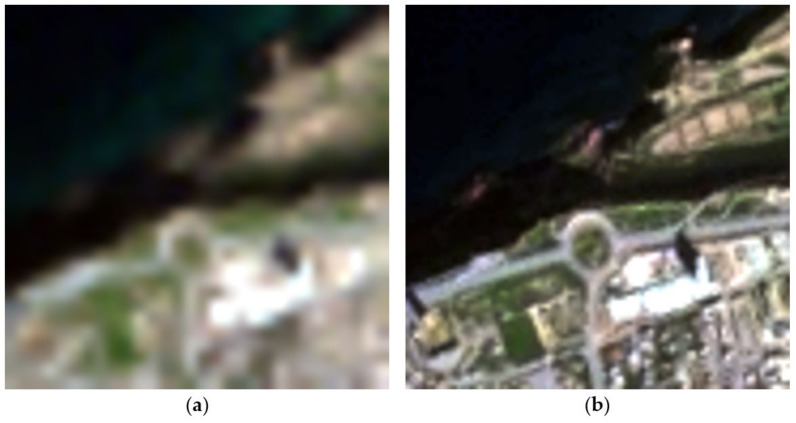
True-color image composite for the real dataset. (**a**) Low-spatial-resolution hyperspectral image; (**b**) High-spatial-resolution pansharpened multispectral image.

We also considered real data for the conducted experiments, specifically a real hyperspectral and a real multispectral image. These two images were acquired on the same day (3 March 2003) and at the same time [40,59]. These real data were geometrically coregistered and radiometrically corrected and cover a small part of the urban area of Oran (Algeria). These images were mainly composed of seven classes of endmembers. The low-spatial-resolution hyperspectral image was acquired by the Earth Observing-1 (EO-1) [2] Hyperion sensor with 125 spectral bands and 30×30 pixels (Figure 2a). This hyperspectral image presented a spatial resolution of 30 m. The high-spatial-resolution pansharpened multispectral image was acquired by the EO-1 Advanced Land Imager (ALI) with 9 spectral bands (Table 2) and 90×90 pixels (Figure 2b). The multispectral image had a 10 m spatial resolution which represents a scale factor of three between the hyperspectral and multispectral images.

## 5. Experiments

### 5.1. Performance Criteria

To evaluate the performance of the proposed method and the tested state-of-the-art methods, various metrics were employed. For the synthetic data set, the obtained sharpened hyperspectral products $\tilde{X_{f}}$ from the introduced method and the tested state-of-the-art techniques were compared to the reference image X using spectral and spatial performance criteria. The first quality measure for the synthetic data is the *Spectral Angle Mapper* (*SAM*). The *SAM* at the *i*-th pixel is obtained as follows [77]

(9) $$SAM_{i}=arcos\;\left(\frac{\langle x_{i},\;\;\tilde{x_{f_{\dot{i}}}}\rangle }{∥x_{i}{∥}_{2}.∥\tilde{x_{f_{\dot{i}}}}{∥}_{2}}\right)$$

with i=1 ⋯P. The average value of the *SAM* over all pixels was used to determine the quality of the fusion product. The lower the value of the *SAM*, the better the method.

The second performance criterion was the Spectral Normalized Mean Square Error $NMSE_{\lambda }$ [59]

(10) $$NMSE_{\lambda_{i}}=\frac{∥x_{i}-\tilde{x_{f_{\dot{i}}}}{∥}_{2}}{∥x_{i}{∥}_{2}^{}}$$

As for the *SAM*, the average value of the $NMSE_{\lambda }\;$ over all pixels was used to determine the spectral quality of the fusion product. The ideal value for the $NMSE_{\lambda }$ is 0.

The third performance criterion was the Spatial Normalized Mean Square Error $NMSE_{S}$ [59]

(11) $$NMSE_{s_{k}}=\frac{∥X_{k}-\tilde{X_{f_{K}}}{∥}_{2}}{∥X_{k}{∥}_{2}}$$

where $X_{k}$ and ${\tilde{X}}_{f_{k}}$ are the *k*-th spectral band of the reference hyperspectral image and the estimated sharpening product. The average value of the $NMSE_{S}$ over all spectral bands was used to determine the spatial quality of the fusion product. The ideal value for the $NMSE_{S}$ is 0.

We also used the Erreur Relative Globale Adimensionnelle de Synthèse (ERGAS) defined in [77]

(12) $$ERGAS=100r\sqrt{\frac{1}{L_{h}}\overset{L_{h}}{\underset{l=1}{\sum }}{\left(\frac{RMSE_{l}}{\mu \left(\tilde{X_{f_{l}}}\right)}\right)}^{2}}$$

where r is the spatial factor between MSI and HSI, $\mu \left(\tilde{X_{f_{l}}}\right)$ the mean of the estimated image and $RMSE_{l}$ represents the *Root Mean Square Error*. The ideal value for the *ERGAS* is 0.

The *Peak Signal to Noise Ratio* (*PSNR*) was also used [77]

(13) $$PSNR_{l}=20\times log_{10}\left(\frac{max\left(\tilde{X_{f_{l}}}\right)}{RMSE_{l}}\right)$$

where $RMSE_{l}$ represents the *Root Mean Square Error*. A higher value of PSNR means a better spatial reconstruction.

The last quality metric used for the synthetic data was the *Universal Image Quality Index* (*UIQI*) [77]

(14) $$UIQI\left(X\;,\;{\tilde{X}}_{f}\right)=\frac{4\;\delta_{X\;{\tilde{X}}_{f}}\;.\;\mu \left(X\right)\;.\;\mu \left({\tilde{X}}_{f}\right)}{\left(\sigma_{X}^{2}+\sigma_{{\tilde{X}}_{f}}^{2}\right)\;\left(\mu {\left(X\right)}^{2}+\mu {\left({\tilde{X}}_{f}\right)}^{2}\right)}$$

where $\delta_{X\;{\tilde{X}}_{f}}$ is the covariance between the reference image X and the estimated image $\tilde{X_{f}}$. $\sigma_{X}^{2}$ and $\sigma_{{\tilde{X}}_{f}}^{2}$ are their variances. μ(X) and $\mu \left(\tilde{X_{f}}\right)$ denote their means. The *UIQI* varies between −1 and 1. Its ideal value is 1 which indicates a perfect reconstruction.

For the real data, other types of metrics were used as there is no ground truth for such data. The *Modified Quality with no Reference* criterion (*mQNR*) [40] was considered. The *mQNR* is based on the *Quality with no Reference* (QNR) [78] and was modified to incorporate the hypersharpening process. The mQNR is given by

(15) $$mQNR={\left(1-D_{\lambda }\right)}^{\sigma }{\left(1-D_{s}\right)}^{\rho }$$

where σ and ρ are real-valued exponents set to 1 for the test conducted on real data. $D_{S}$ and $D_{\lambda }$ represent the spatial and spectral distortion indices. The spectral distortion index $D_{\lambda }$ reads [40]

(16) $$D_{\lambda }=\sqrt[{\omega }]{\frac{1}{L_{h}\left(L_{h}-1\right)}\sum_{j=1}^{L_{h}}\sum_{r=1,\;r\neq j}^{L_{h}}{\left|\mathrm{UIQI}\left({\tilde{X}}_{f_{j}},{\tilde{X}}_{f_{r}}\right)-\mathrm{UIQI}\left(X_{h_{j}},X_{h_{r}}\right)\right|}^{\omega }}$$

where ω is a positive exponent set to 1 for the experiments. $X_{f_{*}}$ is a spectral band of the fusion product and $X_{h_{*}}$ is a spectral band of the reference hyperspectral image.

The spatial distortion index $D_{s}$ was obtained as follows. A subindex was first calculated as mentioned in [78] between each multispectral band and hyperspectral bands covered by the same multispectral band without considering the hyperspectral bands outside the spectral range of the multispectral image. The final spatial distortion index $D_{s}$ is the mean of the estimated subindices.

### 5.2. Results and Discussion

#### 5.2.1. Results for Synthetic Dataset

The first part of the tests was devoted to the synthetic dataset. The regularization parameters, for HMF-IPNMF [59,65], HySure [41,77] and FuVar [58], considered for the synthetic dataset are reported in Table 3.

For the HMF-IPNMF method, the maximum number of iterations of IP-NMF was set to 100. For the CNMF method, the numbers of iterations for the inner and outer loops were fixed to 100 and 3, respectively. For the FuVar and HySure methods, the blurring kernel was assumed to be known a priori. As an initialization step for all the state-of-the-art methods, the VCA method was applied. The FCLS method was used to initialize the abundance coefficients for the FuVar CNMF and HMF-IPNMF methods. As the CNMF and HySure methods do not consider the spectral variability, they were executed by fixing the number of pure materials to 30 as suggested in [77]. This allows them to have more flexibility and to somehow manage the spectral variability although they are not designed for it. The HMF-IPNMF and FuVar methods were applied by considering the manually determined number of classes of endmembers equal to 7, because these two methods deal with the spectral variability.

For the HSB-SV method, all the fixed parameters are reported in Table 4. The numbers of pure materials and subsets were fixed to perform the spectral reconstruction while preserving the processing time as much as possible.

**Figure 3:**
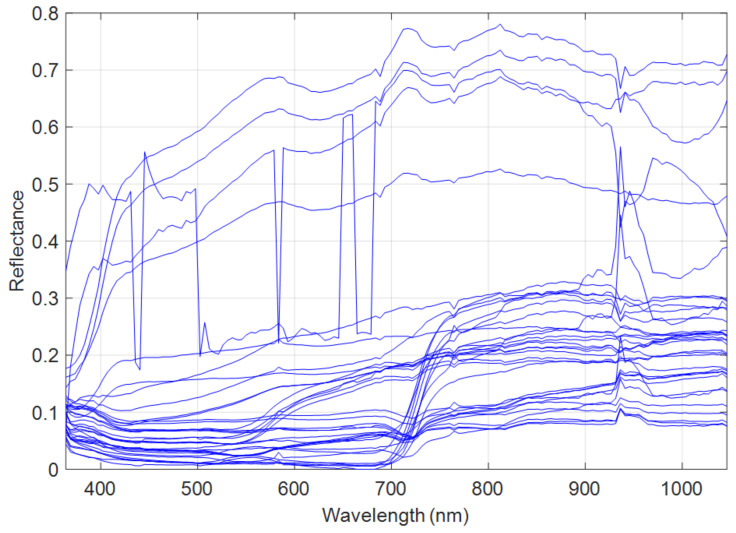
Extracted spectral library from the synthetic data by AEEB.

Finally, the CPU used in the conducted experiments was an Intel Core i5-8350U processor running at 1.70 GHz, with a memory capacity of 16 GB. The results of the quality metrics for the synthetic dataset are reported in Table 5. Figure 3 illustrates the spectra library extracted from the HSI by means of the AEEB method. Figure 3 clearly shows the presence of spectral variability and corroborates that this phenomenon must be taken into account.

Table 5 clearly shows that the methods considering the spectral variability yielded the best results, in particular HMF-IPNMF and HSB-SV. The FuVar method reached the highest value of NMSE_λ_ with 14.42% which led to the worst spectral reconstruction in terms of spectral fidelity compared to the tested methods. This finding demonstrates that the spectral variability is not induced by only illumination or topography changes in the observed scene. The CNMF and HySure methods improved this aspect by obtaining an NMSE_λ_ of 11.89% and 9.62%, respectively. The HMF-IPNMF method provided the best spectral reconstruction for the tested state-of-the-art methods with a value of SAM of 3.53° and NMSE_λ_ equal to 7.92%. The HSB-SV method delivered the best overall results denoted by spectral performance criteria with the lowest values of the SAM (2.65°) and the NMSE_λ_ (7.49%), which clearly demonstrates the superior performance of HSB-SV in terms of spectral fidelity compared to the tested state-of-art-methods. These findings show that considering a spectral library to estimate the hyperspectral spectra is very effective.

**Figure 4:**
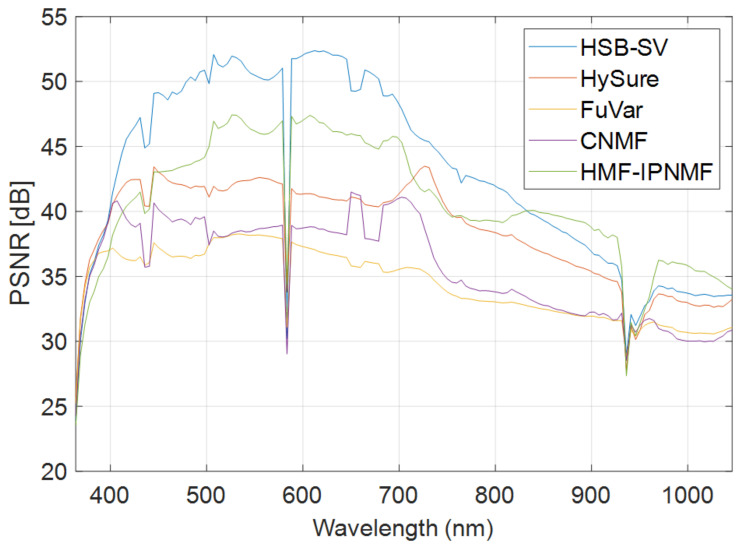
Band-wise PSNR for the synthetic dataset.

Furthermore, these findings were confirmed by the PSNR with the highest value (equal to 43.01) corresponding to a substantial gain of nearly 3 dB as compared with HMF-IPNMF and 10 dB with respect to the FuVar method. To illustrate the notable gain of HSB-SV, Figure 4 represents the PSNR of all methods for each spectral band for the synthetic dataset. This figure illustrates clearly that the HSB-SV method obtains the best values of the PSNR in almost all the spectral bands, particularly between band 20 and band 80.

The spatial quality metrics confirmed the findings obtained from the spectral metrics, the HSB-SV method outperforms the other methods in terms of spatial fidelity, with the highest value of the UIQI and the lowest value of the ERGAS. These results clearly show the attractiveness of using sparse regression to estimate high-spatial-resolution abundance fractions. Indeed, the AEEB method provides a quite suitable spectral library which improves the performance of the SUnSAL method.

These results demonstrate that using a spectral library improves the spectral reconstruction on one hand and it enhances the spatial fidelity on the other hand by providing SUnSAL with a suitable spectral library.

The processing times of the tested techniques are reported in Table 6. As expected, as the HMF-IPNMF and FuVar methods consider matrices with large sizes to tackle the spectral variability, their processing times were the highest with running times equal to 465.98 s and 363.64 s, respectively. The HSB-SV method achieved the best results with the lowest execution times when compared to the tested state-of-art methods. It should be noted that the high-quality spectral and spatial reconstructions obtained by the HSB-SV method do not come at the price of a higher processing time as it is reported in Table 6. Indeed, although the HSB-SV method deals with spectral variability like the HMF-IPNMF and FuVar methods, HSB-SV provided the best results with a computation time equal to 0.74 s. Moreover, the HSB-SV method had a lower computing time compared to the CNMF and HySure methods (with computational times around 3.20 s and 12.89 s, respectively) even though these methods are not considering spectral variability and are supposed to be more efficient in terms of computational cost. These findings prove the effectiveness of the HSB-SV method to deal with spectral variability during the hypersharpening process by providing products with high fidelity reconstruction at a very low processing cost. This significant reduction of the processing time is caused by two main elements. The first one is that the AEEB method is very efficient, in particular a fast algorithm (VCA) is considered for the extraction of endmembers. The second main reason is the efficiency of the SUnSAL method.

**Figure 5:**
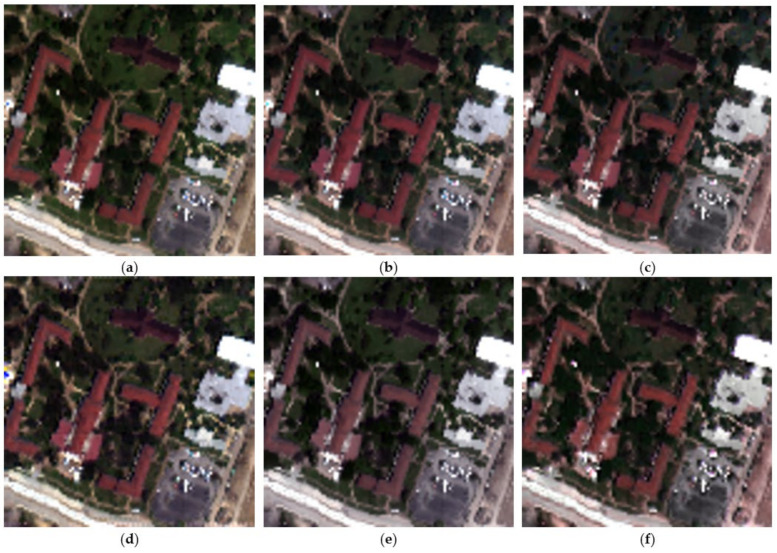
True-color image composite for the synthetic dataset. (**a**) Original hyperspectral image; (**b**) Obtained HSB-SV sharpened hyperspectral image; (**c**) Obtained HMF-IPNMF sharpened hyperspectral image; (**d**) Obtained HySure sharpened hyperspectral image; (**e**) Obtained CNMF sharpened hyperspectral image; (**f**) Obtained FuVar sharpened hyperspectral image.

For the visual inspection of the obtained results, Figure 5 illustrates the true color composite of the synthetic dataset for all tested methods. We can observe clearly that the HSB-SV and HMF-IPNMF methods achieved the best spatial reconstruction when compared to the other methods, particularly compared to CNMF and FuVar, which have many spatial and spectral distortions present in the buildings area (red buildings).

**Figure 6:**
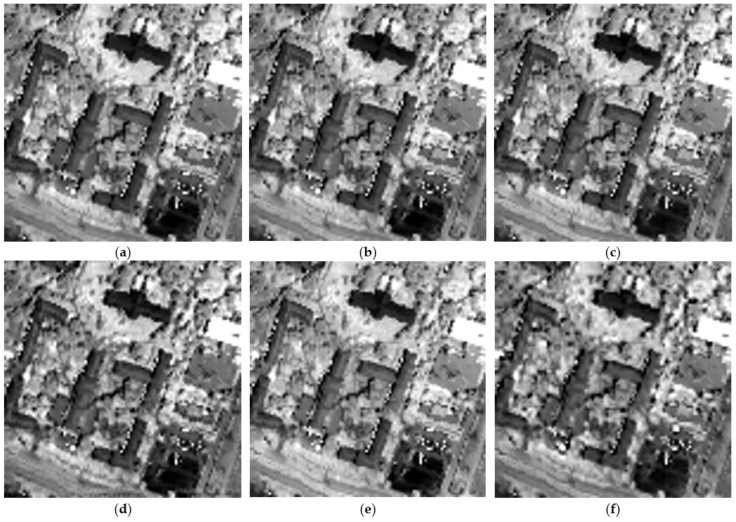
Spectral band in the 0.850 μm region (**a**) Original hyperspectral image; (**b**) Obtained HSB-SV sharpened hyperspectral image; (**c**) Obtained HMF-IPNMF sharpened hyperspectral image; (**d**) Obtained HySure sharpened hyperspectral image; (**e**) Obtained CNMF sharpened hyperspectral image; (**f**) Obtained FuVar sharpened hyperspectral image.

To have a clearer view of the spatial gain obtained by the HSB-SV method (as it is difficult to make a clear conclusion from the color composite images), Figure 6 shows the obtained hypersharpening products for all the methods for the spectral band in the 0.850 μm region. This figure clearly shows that the HSB-SV method comes with the lowest spatial distortions compared to the other tested methods and it is the closest to the reference hyperspectral image.

#### 5.2.2. Results for Real Dataset

**Figure 7:**
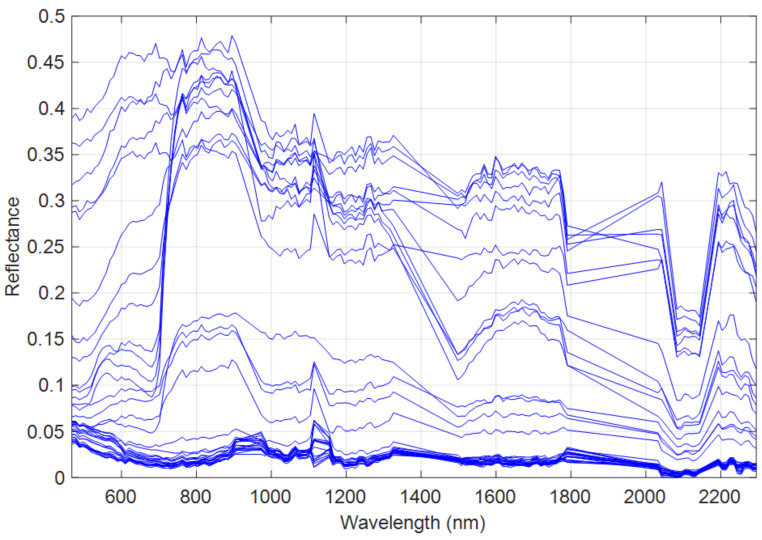
Spectral library extracted from the real data by AEEB.

The second part of the tests is devoted to the real data. The regularization parameters were identical to those in the tests performed with the synthetic dataset for the state-of-the-art methods (Table 3). For the HSB-SV method, the used parameters are reported in Table 7. The spectra library extracted from HSI by means of the AEEB method is illustrated in Figure 7. Figure 7 clearly illustrates the presence of spectral variability in the real dataset and thus the notion of class of endmembers must be considered.

Table 8 reports the performance metrics for the real dataset. The CNMF method obtained the highest values for $D_{\lambda }$ and $D_{s}$ showing that it provided the lowest spectral and spatial fidelities of this benchmarking. The FuVar method improved this aspect with a better spatial and spectral reconstruction as it considered spectral variability. The HMF-IPNMF method achieved the best overall results among the tested state-of-the-art methods in particular for the mQNR above 0.95 which proves that it handles the spectral variability better than the FuVar method (equal to 0.9288). The HSB-SV method outperformed all the other tested methods in terms of spectral and spatial fidelities. In particular, it achieved the best spatial reconstruction with $D_{s}$ equal to 0.0064. This spatial enhancement was mainly due to the SUnSAL method, which was applied by considering a suitable spectral library. Furthermore, the HSB-SV method obtained the best value of the mQNR, equal to 0.9615. This finding proves that using a spectral library significantly improves the performance of spectral reconstruction with respect to the tested state-of-art methods.

The execution times for the methods applied to the real data are provided in Table 9. The HSB-SV method was significantly faster compared to the tested state-of-the-art methods and particularly the approaches tackling the spectral variability. As the HMF-IPNMF and FuVar methods are handling the spectral variability with modified LMMs involving matrices with large sizes, they came with the highest processing times, around 461.91 s (HSB-SV was about 2310 times faster than HMF-IPNMF) and 238.99 s (HSB-SV was around 1195 times faster than FuVar), respectively. The CNMF and HySure methods improved this aspect with running times around 1.63 s and 12.02 s, respectively, because they do not consider the spectral variability. However, the CNMF and HySure methods are slower compared to the HSB-SV method, in particular HySure (HSB-SV is around 60 times faster than HySure). The HSB-SV method presented the lowest execution time, below one second, i.e., around 0.20 s. Furthermore, the HSB-SV method brings obvious improvements in terms of spectral and spatial fidelities compared to the methods dealing with spectral variability. It provided a high fidelity with the lowest computational time. These significant improvements with respect to the state-of-the-art tested methods prove the attractiveness of using sparse regression to achieve the best spatial reconstruction at the lowest computational cost.

**Figure 8:**
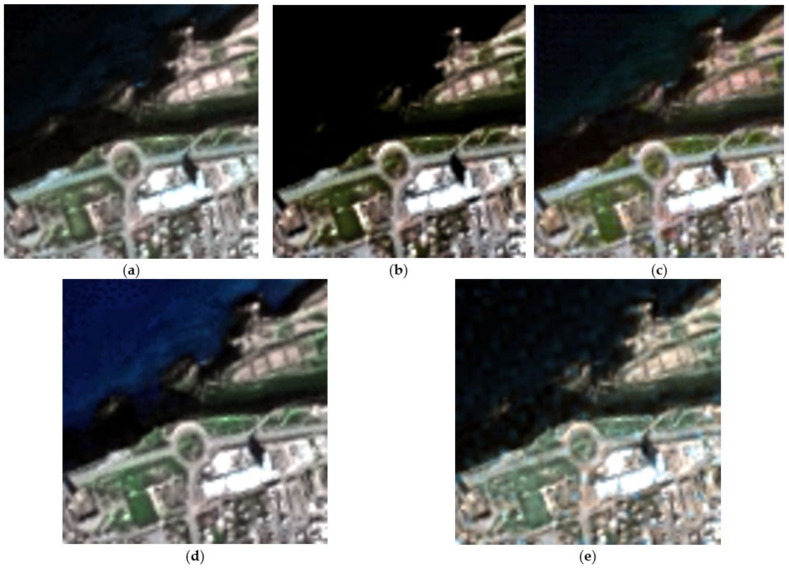
True-color image composite for fusion products derived for the real dataset. (**a**) Obtained HSB-SV sharpened hyperspectral image; (**b**) Obtained HMF-IPNMF sharpened hyperspectral image; (**c**) Obtained HySure sharpened hyperspectral image; (**d**) Obtained CNMF sharpened hyperspectral image; (**e**) Obtained FuVar sharpened hyperspectral image.

To illustrate the performance of the tested techniques, Figure 8 shows the true color composite of the obtained hypersharpening products. It can be seen clearly that the CNMF method presented more spatial distortions in the urban area in particular for the roads (roundabout region). The HMF-IPNMF and HySure methods improved this aspect with less spatial distortion and a better spatial fidelity for the urban area (roundabout region). The HSB-SV method achieved the best spatial reconstruction, in particular the roads were reconstructed with more spatial fidelity.

**Figure 9:**
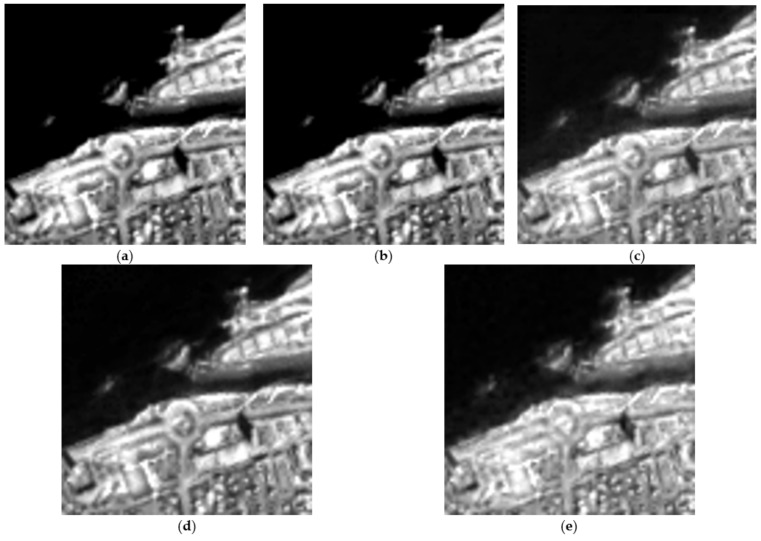
Spectral band in the 0.854 μm region. (**a**) Obtained HSB-SV sharpened hyperspectral image; (**b**) Obtained HMF-IPNMF sharpened hyperspectral image; (**c**) Obtained HySure sharpened hyperspectral image; (**d**) Obtained CNMF sharpened hyperspectral image; (**e**) Obtained FuVar sharpened hyperspectral image.

To have a better visual interpretation of the results, Figure 9 shows the obtained hypersharpening products for all the methods for the spectral band in the 0.854 μm region. This figure demonstrates clearly that the HySure and HSB-SV methods obtained hypersharpening products with the best spatial reconstruction. In particular, the HSB-SV method provided a hypersharpening product with less spatial distortion. Finally, the HSB-SV method proves the effectiveness of the use of the spectral library with sparse regression to achieve the fusion of hyperspectral and multispectral images with both high spatial and spectral fidelities. Indeed, the HSB-SV method allows one to have a hypersharpening product at a very low computational cost compared to methods tackling the spectral variability like HMF-IPNMF and FuVar.

## 6. Conclusions

In this paper, a new hypersharpening method called *Hyperspectral Super-resolution with Spectral Bundles dealing with Spectral Variability* (HSB-SV) is introduced. This technique is related to spectra bundles, more precisely to the *Automated Extraction of Endmember Bundles* (AEEB) method. The AEEB method tackles the spectral variability by constructing a spectral library directly from the hyperspectral image by means of an *Endmember Extraction Algorithm* (EEA) applied to random subsets of the HSI. This straightforward and efficient approach allows one to have a spectral dictionary. Furthermore, it substantially reduces the number of manipulated variables when compared to the hypersharpening methods from the literature which treat the spectral variability. This directly impacts the execution time by reducing it significantly compared to the HMF-IPNMF and FuVar methods, which constitutes the main originality of this work. Indeed, the use of the *Sparse Unmixing by Variable Splitting and Augmented Lagrangian* (SUnSAL) method proves to be very attractive to estimate high-spatial-resolution abundance coefficients while preserving the processing time to a very low level.

The proposed technique was tested on synthetic and real datasets along with some recent state-of-the-art methods. The results, based on spatial and spectral performance criteria, show that the introduced strategy is very attractive and efficient in terms of spectral and spatial reconstructions. The new method outperforms the approaches tested in this paper. Besides, the HSB-SV method has the lowest processing time compared to the considered tested state-of-the-art methods, specifically compared to techniques dealing with spectral variability. These findings prove that using a spectral library appears very effective for the hypersharpening process. Indeed, the AEEB method allows one to construct a spectral library which considers different types of spectral variabilities present in the observed hyperspectral scenes which improves the spectral performance. Moreover, the HSB-SV method enables one to achieve a sufficient reconstruction while providing the lowest execution time of the benchmark. This is mainly due to the efficiency of the SUnSAL method. Moreover, the SUnSAL method yields a sufficient spatial reconstruction quality proving the attractiveness of sparse regression. Indeed, these findings show clearly that the HSB-SV method can handle a complex phenomenon like the spectral variability and still provides good satisfactory results while preserving computational complexity.

An interesting extension of this work may consist of developing techniques considering other spare regression methods to improve the obtained spatial reconstruction. Moreover, future work will focus on improving the efficiency of the AEEB method by reducing the number of pure elements needed to achieve a sufficient spectral reconstruction.

## Figures and Tables

**Table 1:** Spectral bands of the QuickBird Sensor [75].

**Quick Bird**	Spectral Bands (μm)
	0.45–0.52
	0.52–0.60
	0.63–0.69
	0.76–0.90

**Table 2:** Spectral bands of the EO-1 Advanced Land Imager Sensor [76].

**EO-1 Advanced Land Imager**	Spectral Bands (μm)
	0.433–0.453
	0.450–0.515
	0.525–0.605
	0.630–0.690
	0.775–0.805
	0.845–0.890
	1.200–1.300
	1.550–1.750
	2.080–2.350

**Table 3:** Regularization parameters for the HMF-IPNMF, HySure and FuVar methods.

	Regularization Parameters
HMF-IPNMF	μ=30
HySure	$\lambda_{m}=1$ and $\lambda_{\varphi }=10^{-3}$
FuVar	$\lambda_{m}=1$, $\lambda_{A}=$ $10^{-4}$, $\lambda_{1}=0.01$ and $\lambda_{2}=10,000$

**Table 4:** Considered parameters for HSB-SV.

	Experiment Settings for HSB-SV
Number of classes of pure materials	7
Number of subsets	5
Size of Subsets	10%
Sparsity prompting parameter λ (SUnSAL)	$5\times 10^{-4}$

**Table 5:** Performance criteria for the synthetic dataset.

	HSB-SV	HMF-IPNMF	FuVar	HySure	CNMF
SAM (°)	**2.65**	3.53	3.75	3.63	4.34
NMSE_λ_ (%)	**7.49**	7.92	14.42	8.79	11.89
NMSE_s_ (%)	**6.76**	8.52	15.96	9.62	13.73
PSNR (dB)	**43.01**	40.61	34.12	38.73	35.50
UIQI	**0.9728**	0.9627	0.9098	0.9652	0.9402
ERGAS	**4.96**	5.77	10.26	6.18	8.93

**Table 6:** Time processing of the tested methods (in seconds) for the synthetic dataset.

HSB-SV	HMF-IPNMF	FuVar	HySure	CNMF
**0.74**	465.98	363.64	12.89	3.20

**Table 7:** Considered parameters for HSB-SV for the real dataset.

	Experiment Settings for HSB-SV
Number of pure materials	7
Number of subsets	5
Size of Subsets	10%
Sparsity prompting parameter λ (SUnSAL)	$2\times 10^{-4}$

**Table 8:** Performance criteria for the real dataset.

	HSB-SV	HMF-IPNMF	FuVar	HySure	CNMF
$D_{\lambda }$	**0.0322**	0.0335	0.0485	0.0442	0.1243
$D_{s}$	**0.0064**	0.0119	0.0238	0.0098	0.0863
mQNR	**0.9615**	0.9549	0.9288	0.9464	0.8000

**Table 9:** Time processing for each method for the real dataset (in seconds).

	HSB-SV	HMF-IPNMF	FuVar	HySure	CNMF
Time (s)	**0.20**	461.91	238.99	12.02	1.63

## Data Availability

Not applicable.
